# Supplementary material for: Longer growing seasons will not offset growth loss in drought-prone temperate forests of Central-Southeast Europe
Source: Nat Commun. 2025 Oct 29;16:9535. doi: 10.1038/s41467-025-64568-8 (PMC12572185; doi:10.1038/s41467-025-64568-8)
Supplement: Supplementary file 1 — Supplementary Information [file 41467_2025_64568_MOESM1_ESM.pdf]

Longer growing seasons will not offset growth loss in drought-prone temperate forests of Central-Southeast Europe

Jan Tumajer, Jakub Kašpar, Jan Altman, Nela Altmanová, J. Julio Camarero, Emil Cienciala, Vojtěch Čada, Tomáš Čihák, Jiří Doležal, Pavel Fibich, Pavel Janda, Ryszard Kaczka, Tomáš Kolář, Jiří Lehejček, Jiří Mašek, Radim Matula, Kateřina Neudertová Hellebrandová, Lenka Plavcová, Michal Rybníček, Miloš Rydval, Rohan Shetti, Miroslav Svoboda, Martin Šenfeldr, Pavel Šamonil, Ivana Vašíčková, Monika Vejpustková, Václav Treml

Supplementary section

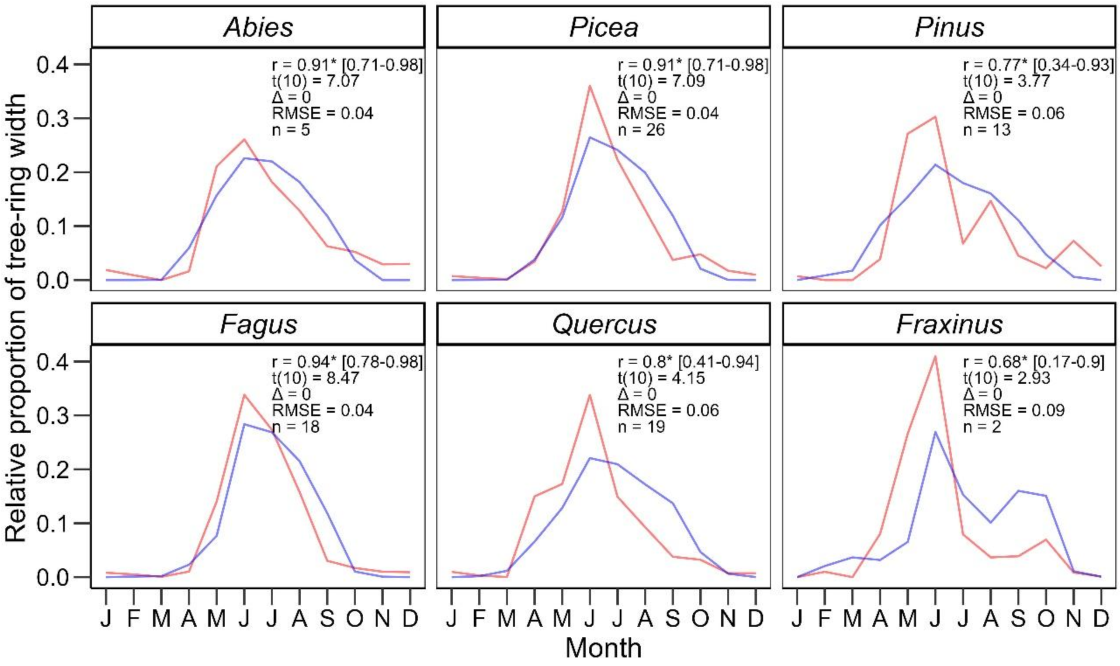

Supplementary Figure 1: Comparison of simulated intra-annual growth dynamics with dendrometer data at genera scale. Mean standardized rate of tree-ring formation from January to December, simulated by the VS-Lite model (blue lines) and recorded by dendrometers (red lines), aggregated for individual genera. The values in the top of each chart indicate goodnes-of-fit statistics between dendrometer data and the simulations (r = Pearson correlation coefficient [its 95 % confidence interval], * = statistically significant correlation according to two-sided test with $p < 0.05$, t(10) = t-value for test with 10 degrees of freedom, RMSE = root mean squared error, Δ = temporal offset in months of seasonal growth peaks) and replication per genera (n = number of independent annual dendrometer observations per genus). Source data are provided as a Source Data file.

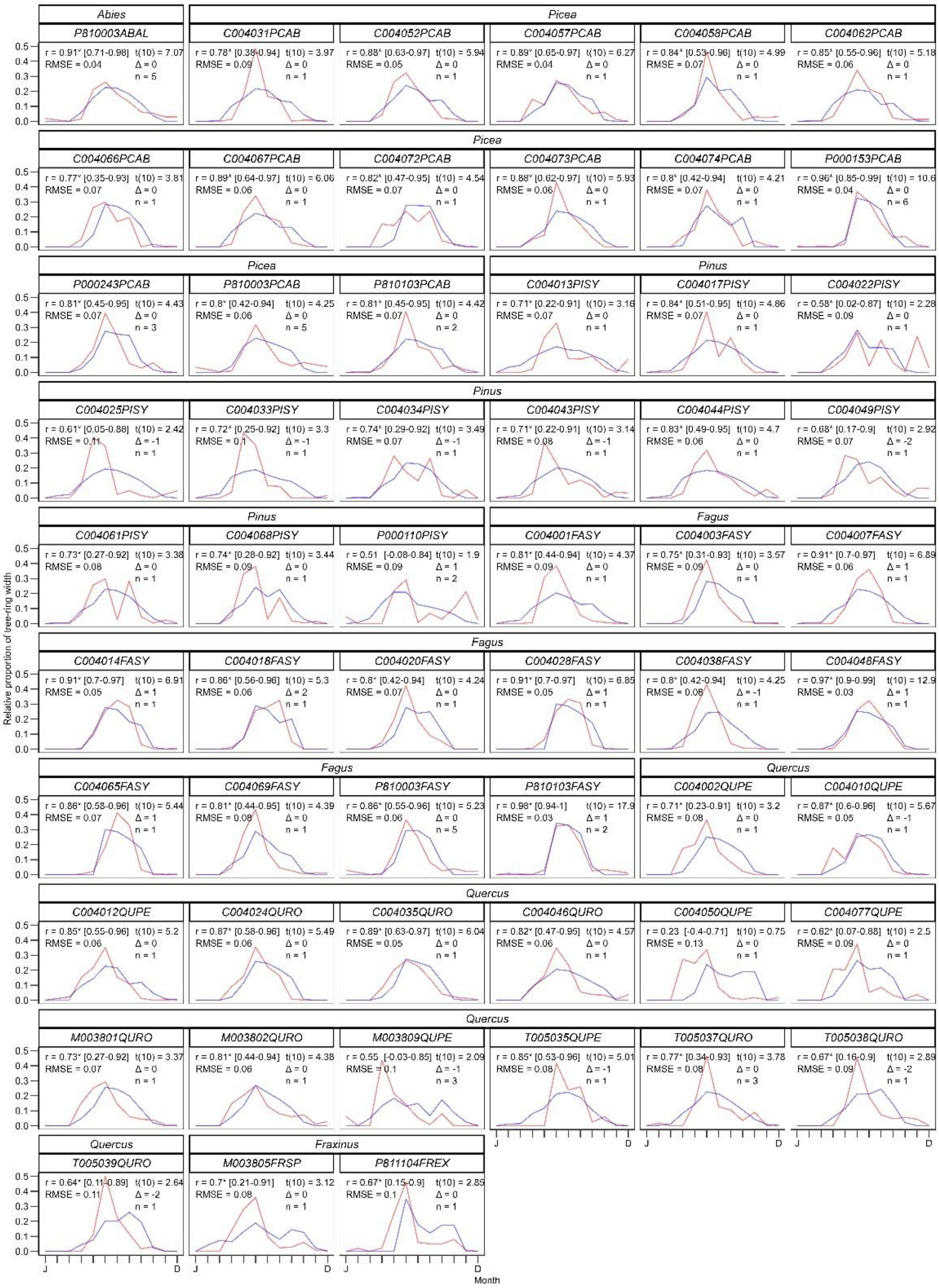

Supplementary Figure 2: Comparison of simulated intra-annual growth dynamics with dendrometer data at site scale. Mean rate of standardized tree-ring formation from January to December, simulated by the VS-Lite model (blue lines) and recorded by dendrometers (red lines) for individual sites. The values in the top of each chart indicate goodnes-of-fit statistics between dendrometer data and the simulations (r = Pearson correlation coefficient [its 95 % confidence interval], * = statistically significant correlation according to two-sided test with $p < 0.05$, t(10) = t-value for test with 10 degrees of freedom, RMSE = root mean squared error, Δ = temporal offset in months of seasonal growth peaks) and sample replication (n = number of independent annual dendrometer observations per site). Source data are provided as a Source Data file.

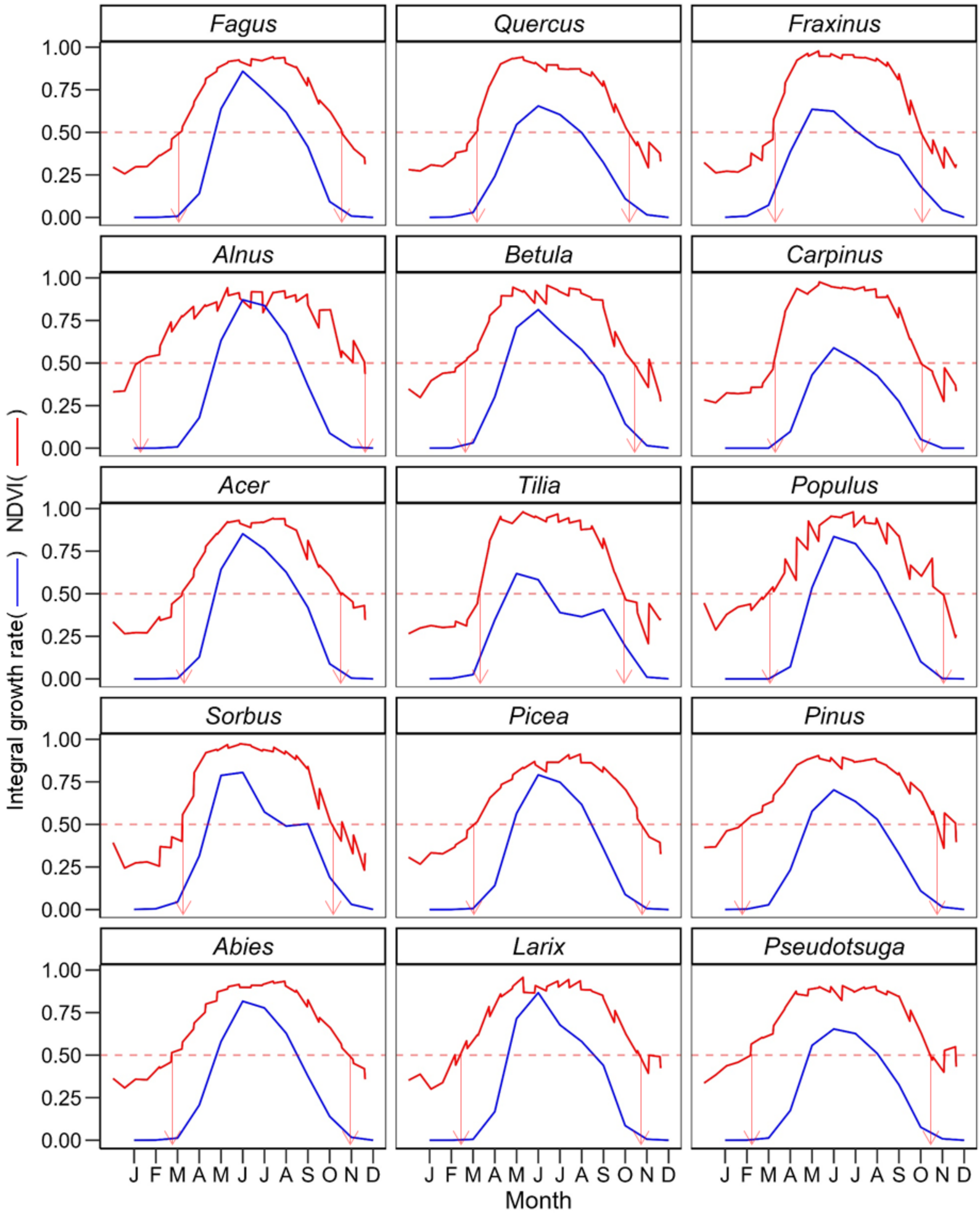

Supplementary Figure 3: **Comparison of simulated intra-annual growth dynamics with NDVI at genera scale.** Mean intra-annual variability of simulated integral growth rates (blue lines) and the standardized Normalized Difference Vegetation Index (NDVI, red lines) for individual genera during 2000-2020. The red arrows highlight the mean date when NDVI exceeded or dropped below 0.5 of its annual amplitude. Source data are provided as a Source Data file.

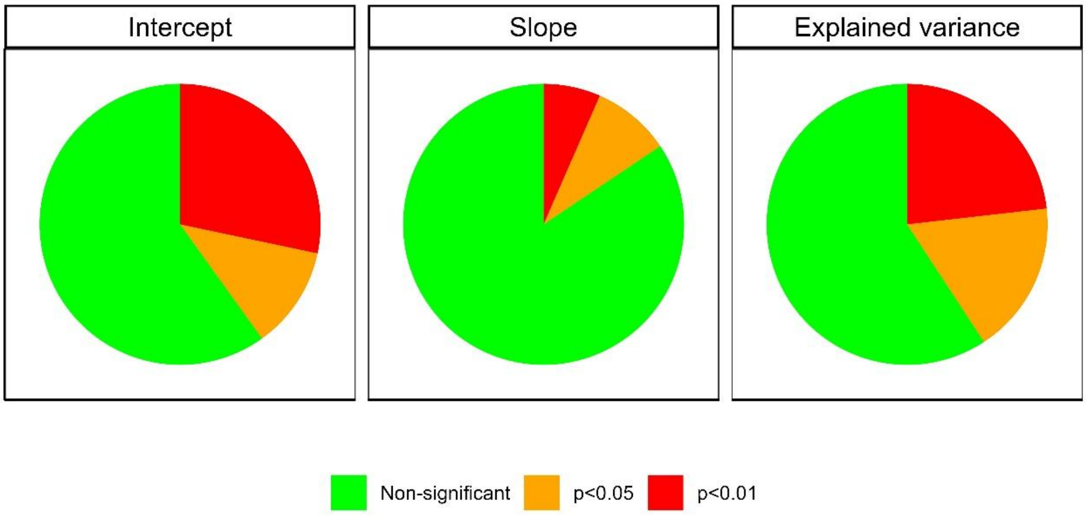

Supplementary Figure 4: **Results of non-stationarity tests.** Proportions of sites with stationary (green) and potentially non-stationary (orange, red) forecasts of the VS-Lite model calculated using the bootstrapped transfer function stability tests during the period from 1961 to the end of the observed site chronology (1995-2020). Note, that the temporal span of chronologies was below the recommended minimum for the application of the bootstrapped transfer function which increases the risk of falsely significant non-stationarity. Source data are provided as a Source Data file.

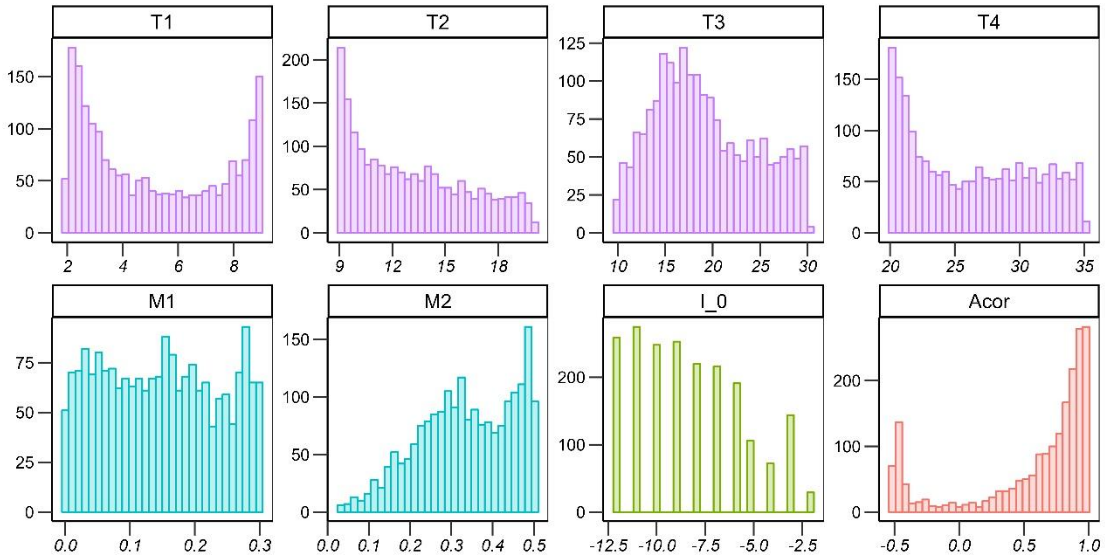

Supplementary Figure 5: Histogram of calibrated parameters of the VS-Lite model (see **Figure 6** for their mechanistic explanation within the model workflow). Source data are provided as a Source Data file.

T1 = Minimum temperature for growth [°C]
T2 = Lower threshold of optimal temperature range [°C]
T3 = Upper threshold of optimal temperature range [°C]
T4 = Maximum temperature for growth [°C]
M1 = Minimum soil moisture for growth [v/v]
M2 = Lower threshold of optimal soil moisture [v/v]
I0 = Start of the integration window in the previous year, i.e., which part of the previous year influences growth of the following year through autocorrelation effect [-]
Acor = Autocorrelation multiplier of previous-year growth rates, i.e., how strong are autocorrelation effects [-]

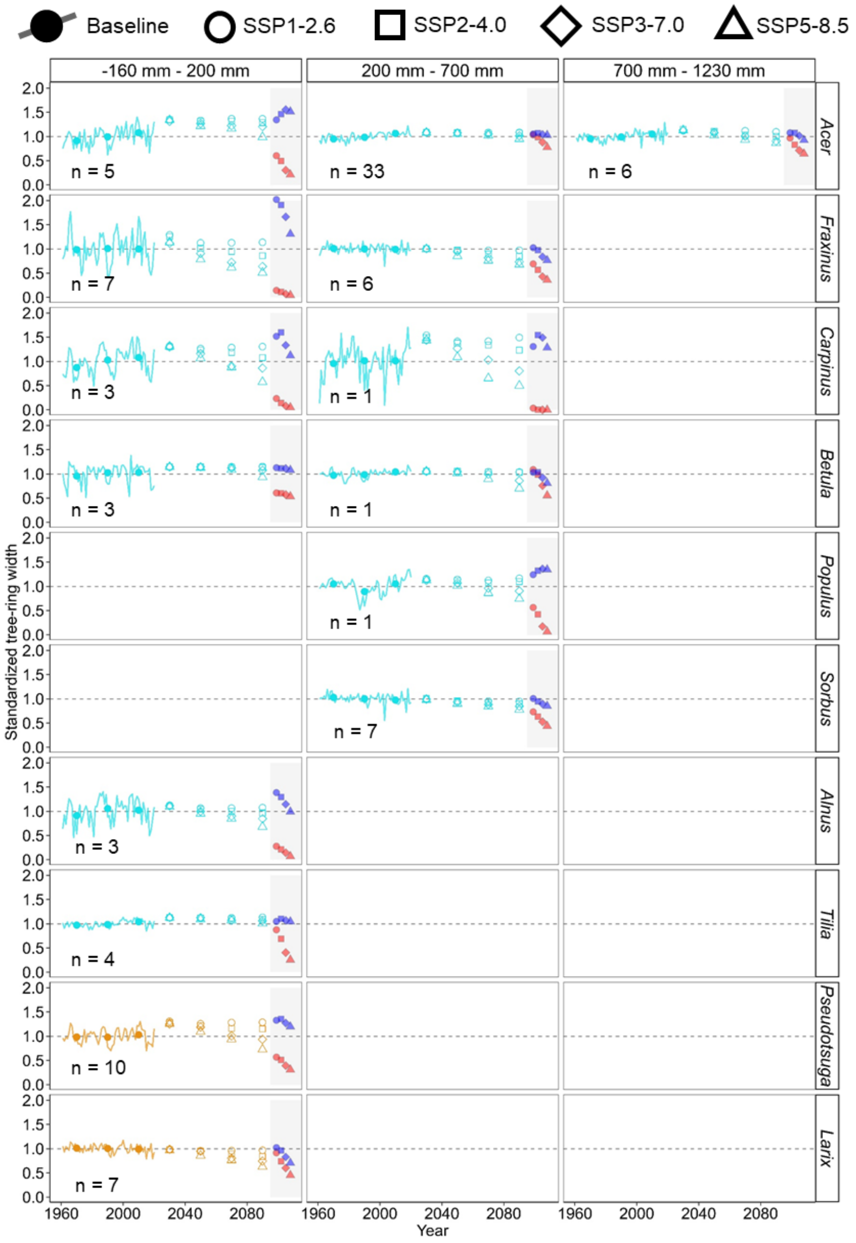

Supplementary Figure 6: **Simulated annual growth increments (i.e., tree-ring width indices)**. Simulated increments are shown for a baseline calibration period 1961-2020 (lines for annual values, full symbols for bi-decadal means) and forecasts for four bi-decadal periods between 2020-2039 and 2080-2099 based on four SSP scenarios of climate change under mean climatic conditions for given bi-decadal period (empty symbols) for rare genera. Full symbols in shaded areas on the right represent annual growth increments during climatic extremes expected by SSP scenarios during 2080-2099 period including wet-cool (blue) and dry-warm extremes (red). Sites were averaged into three belts according to climatic water balance including dry (-160-200 mm), moderate (200-700 mm) and humid (700-1230 mm). n indicates the number of sites for a given range of climatic water balance and genus. Tree-ring width indices were standardized by mean simulated tree-ring widths in the baseline period. Source data are provided as a Source Data file.

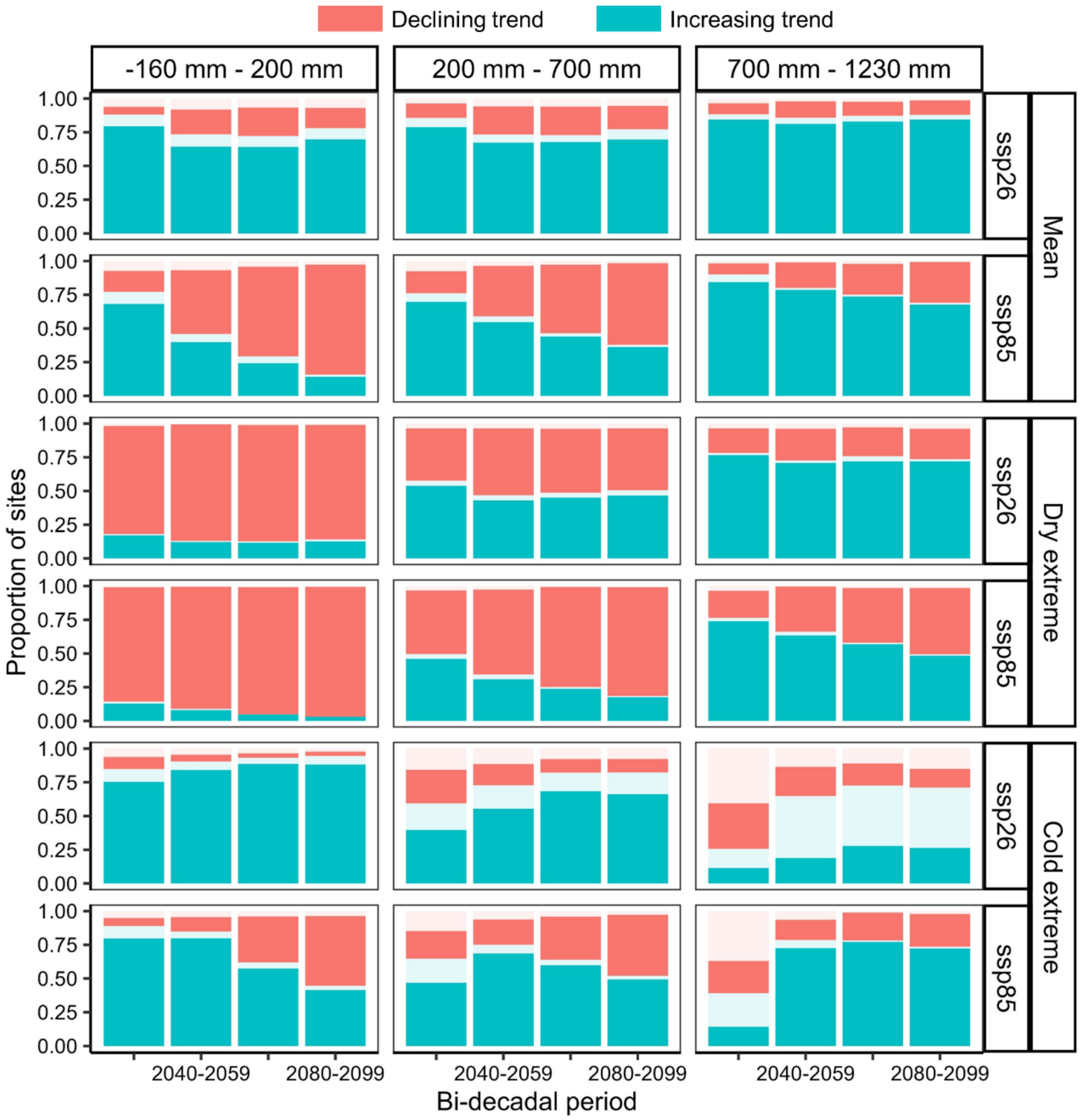

Supplementary Figure 7: Trends in simulated tree-ring widths. Relative frequency of sites with statistically significant (full colors) and non-significant (semi-transparent colors) shifts in forecast tree-ring widths in four bi-decadal periods (x-axes) under selected low-emission (ssp26) and high-emission (ssp85) scenarios from baseline mean. Colors represent tree-ring width increase (blue) and decline (red) in a forecasted period compared to baseline period. Statistical significance was tested by two-sided Student t-test with a level of 0.05. Source data are provided as a Source Data file.

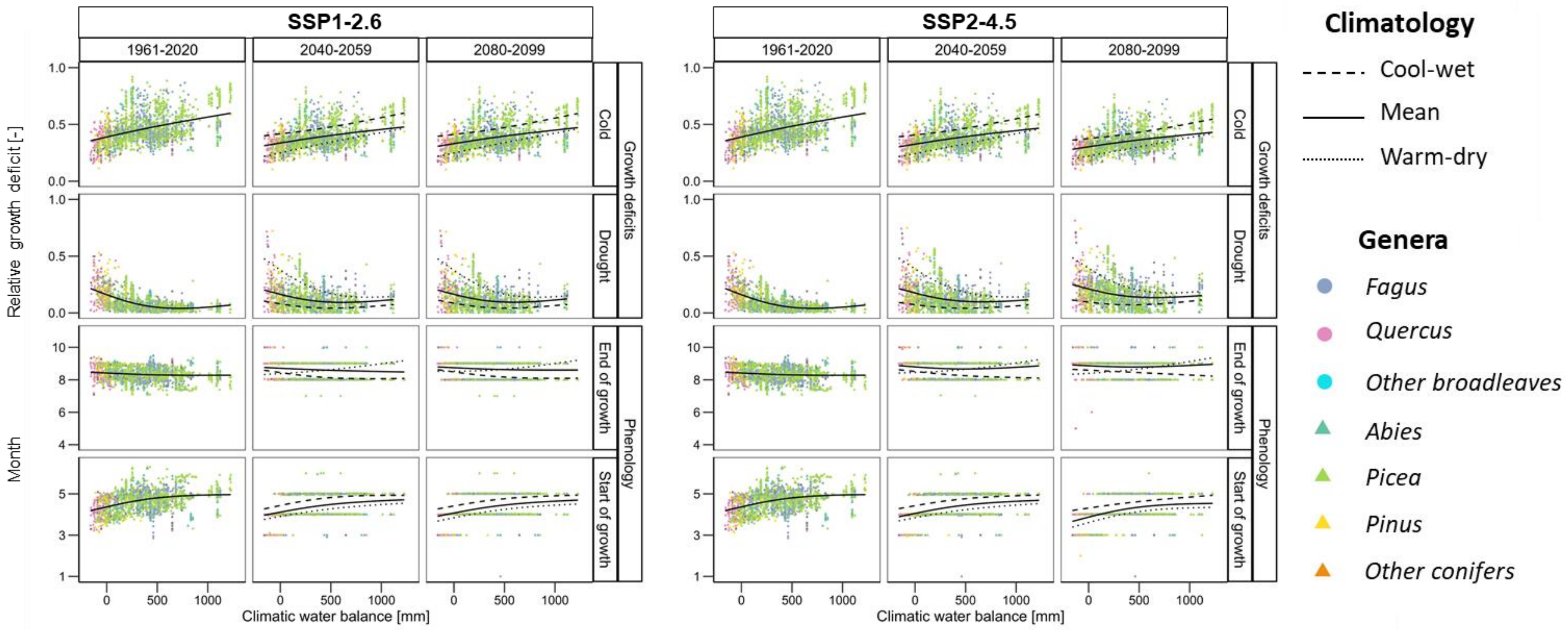

Supplementary Figure 8: Simulated growth deficits and phenology. Mean simulated growth deficits due to drought and cold stress, and timing of growing season start and end for a baseline period 1961-2020, 2040-2059, and 2080-2099 according to two low-emission SSP scenarios. Points show mean values for each site forecasted for each bi-decadal period under mean climatology. Solid lines represent generalized additive models fitted through individual site observations along gradient of climatic water balance. Dashed and dotted lines highlight a shift of generalized additive models if forecasts for climatic extremes within given bi-decadal period were considered instead of the mean climatology. Source data are provided as a Source Data file.

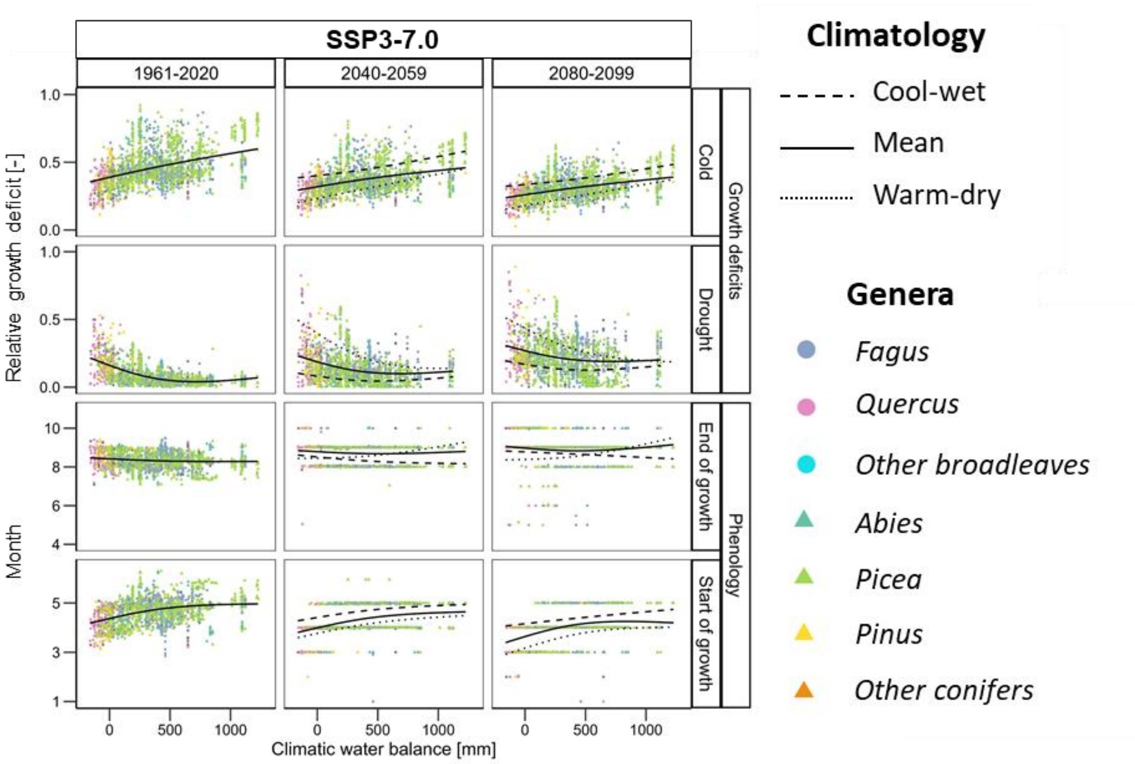

Supplementary Figure 9: Simulated growth deficits and phenology. Mean simulated growth deficits due to drought and cold stress, and timing of growing season start and end for a baseline period 1961-2020, 2040-2059, and 2080-2099 according to high-emission SSP3-7.0 scenario. Points show mean values for each site forecasted for each bi-decadal period under mean climatology. Solid lines represent generalized additive models fitted through individual site observations along gradient of climatic water balance. Dashed and dotted lines highlight a shift of generalized additive models if forecasts for climatic extremes within given bi-decadal period were considered instead of the mean climatology. Source data are provided as a Source Data file.

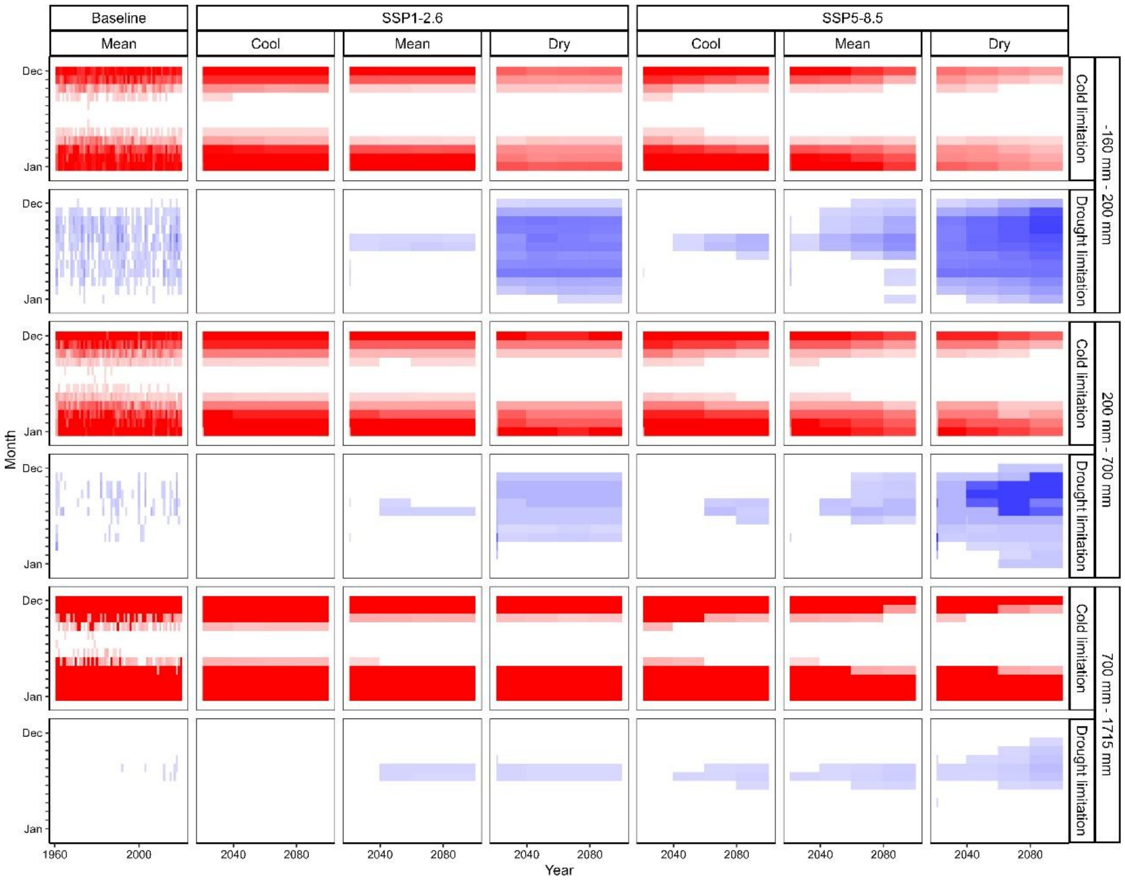

Supplementary Figure 10: **Simulated shifts in growth cessation**. Mean frequency of growth cessation (i.e., occurrence of zero integral growth rate) in specific calendar months (y-axes) and years (x-axes) across all sites with climatic limiting factor driving the growth cessation. Results for baseline period 1961-2020 (left column), low-emission forecast (SSP1-2.6), and high-emission forecast (SSP5-8.5) are shown. For each forecast, simulations are presented for mean and extreme climatologies within given bi-decadal period. White color indicates no occurrence of growth cessation due to given climatic limiting factor, full red and blue colors highlight months when all sites of our network stopped their growth due to given climatic limitation. Source data are provided as a Source Data file.

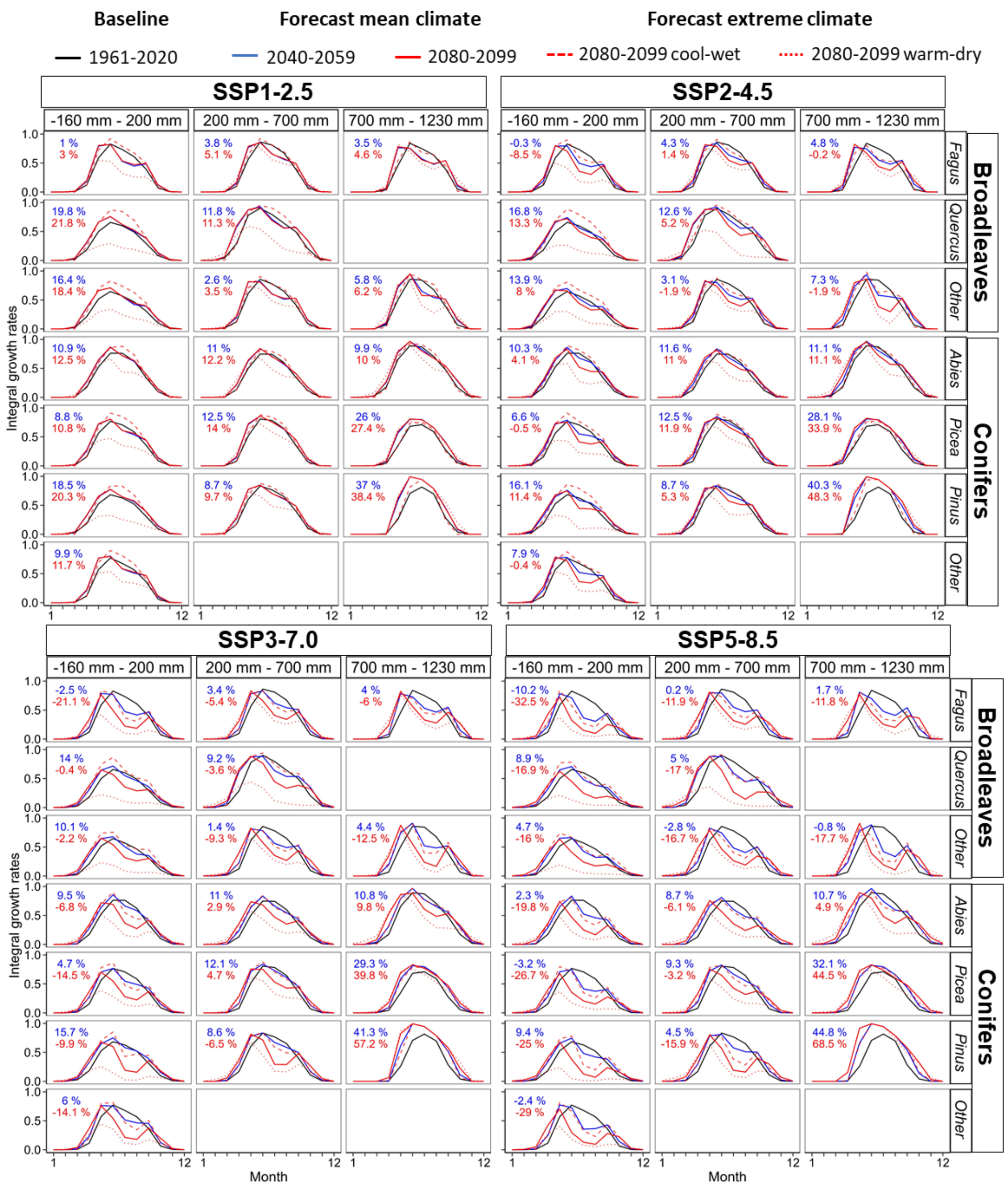

Supplementary Figure 11: **Simulated intra-annual growth patterns.** Mean simulated monthly integral growth rates for the baseline period 1961-2020 (black), 2040-2059 (blue), and 2080-2099 (red) along gradient of climatic water balance reflecting four SSP scenarios. Red lines are based on simulations reflecting mean (solid), extremely cool-wet (dashed), and extremely warm-dry (dotted) years predicted for the 2080-2099 period. Sites were averaged into groups according to climatic water balance in the baseline period including dry (-160-200 mm), moderate (200-700 m), and humid (700-1230 mm). Values in the top-left corner indicate the difference between the annual sum of integral growth rates between forecasts for future decades under mean climatology and the baseline period. Source data are provided as a Source Data file.

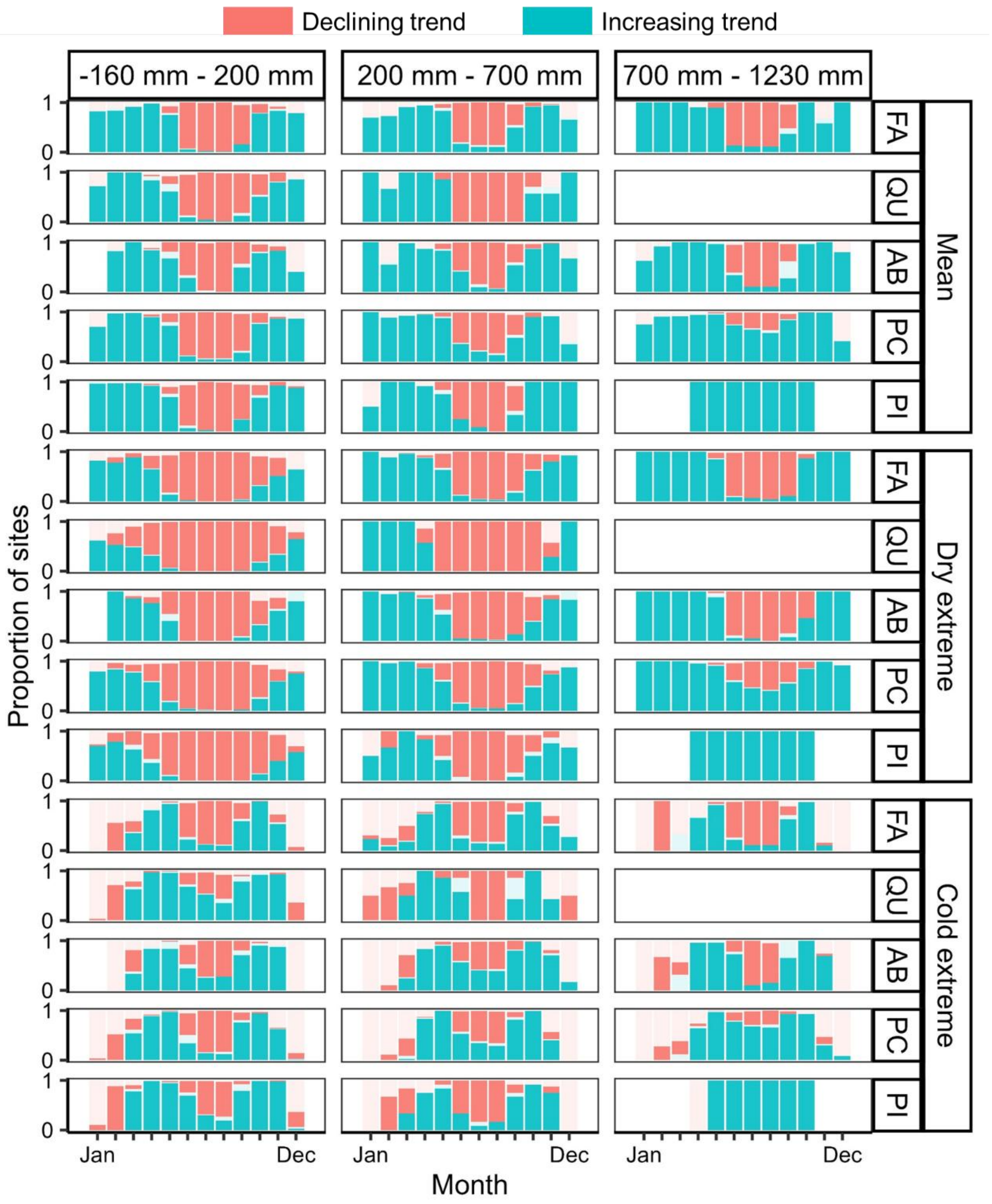

Supplementary Figure 12: **Trends in simulated monthly integral growth rates.** Distribution of sites with significantly (full color) or non-significantly (semi-transparent color), positive (blue) or negative (red) differences in simulated monthly integral growth rates from the baseline period. Results are presented for 2080-2099 period and mean and extreme climatologies based on SSP5-8.5 scenario. Sites are aggregated along the climatic gradient (columns) and main genera (rows): FA = *Fagus* sp., QU = *Quercus* sp., AB = *Abies* sp., PC = *Picea* sp., PI = *Pinus* sp. Statistical significance was tested by Student t-test with level of 0.05. Source data are provided as a Source Data file.

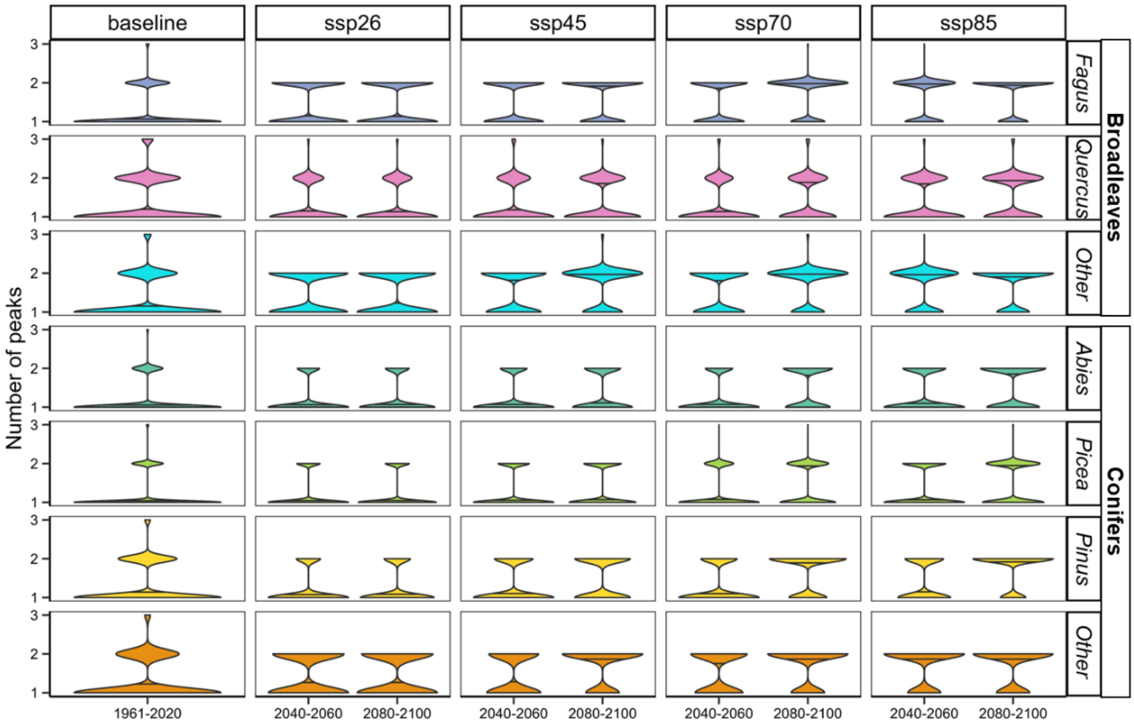

Supplementary Figure 13: **Simulated intra-annual growth uni/bi-modality.** Violin charts for distributions of the numbers of local maxima in integral growth rates per year (i.e., growth peaks during the growing season) simulated by the VS-Lite model in the baseline period, 2040-2059, and 2080-2099 according to four SSP scenarios and mean climatology. The local maximum was defined as a month in which the integral growth rate exceeded the growth rates of both preceding and following months. Horizontal line indicates median of the distribution. Source data are provided as a Source Data file.

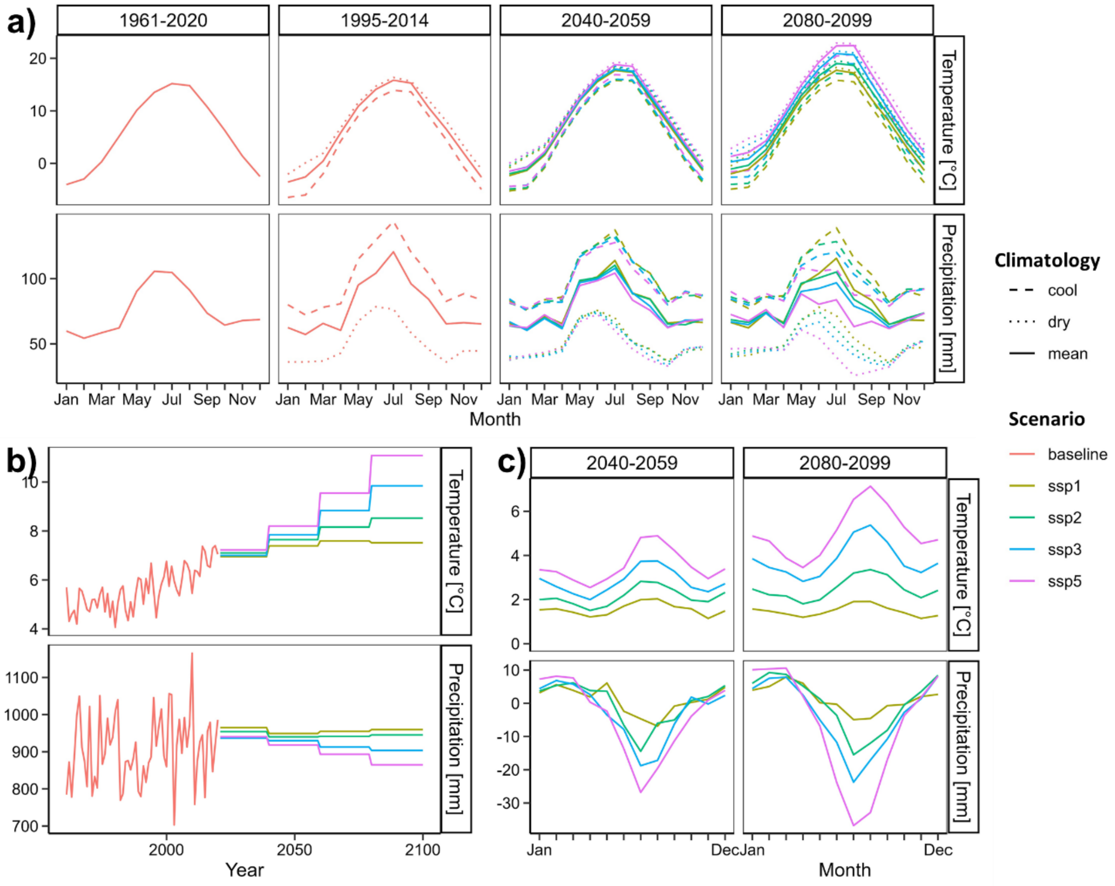

Supplementary Figure 14: Mean historical and forecast climatic data across all sites (a) mean monthly temperatures and precipitation. Baseline means for the calibration period of the VS-Lite model (1961-2020) and the CMIP6 normal (1995-2014) are shown. Forecast climatic data for the 2040-2059 and 2080-2099 were derived from bi-decadal anomalies reflecting four SSP scenarios (colors) and three climatologies of mean, warm-dry, and cool-wet years during normal period (linetypes). (b) Mean annual temperatures and precipitation totals for baseline years and forecast bi-decadal periods. (c) Monthly bi-decadal anomalies of SSP scenarios from 1995-2014 climatic normal. Source data are provided as a Source Data file.

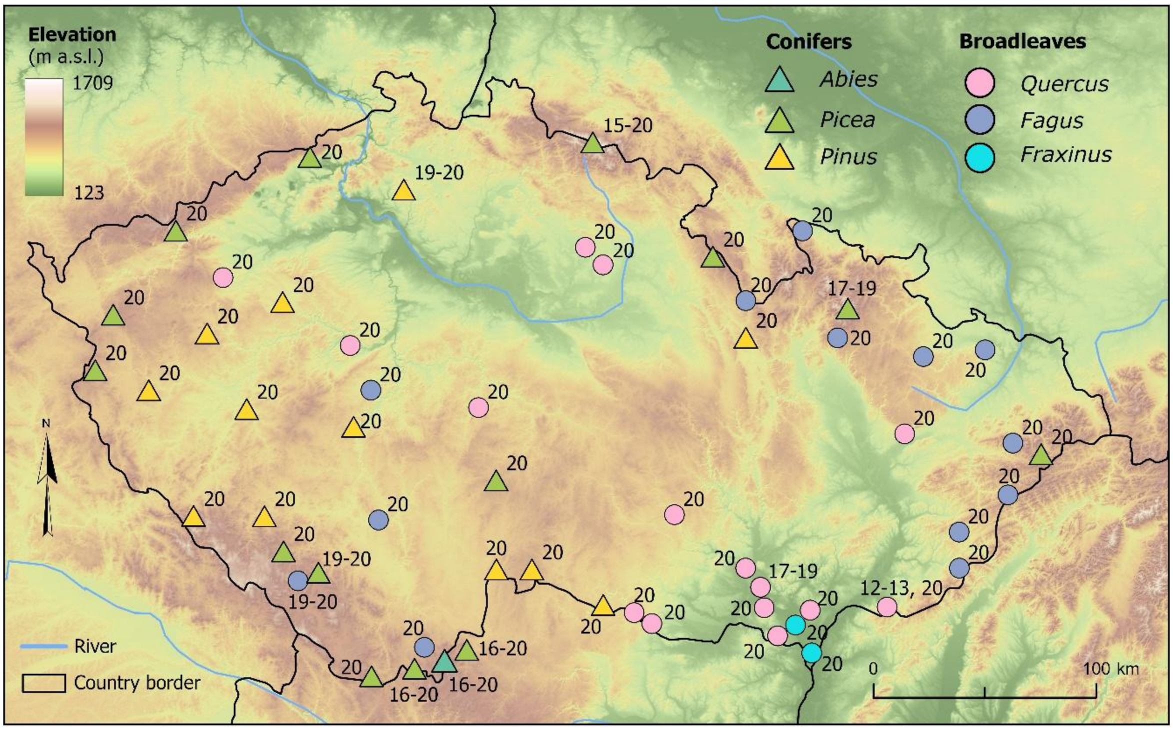

Supplementary Figure 15: Spatial distribution of sites equipped with dendrometers in the Czech Republic. Numbers next to each site code calendar years with available dendrometer data within 2012-2020 period (e.g., 20=2020). The underlying map was created using the Natural Earth database and SRTM digital elevation model (NASA/USGS).

Supplementary Table 1: Species composition of the dendrochronological dataset.

Group	Genera		Species	
	Name	Number of sites	Name	Number of sites
Conifers	*Picea*	1,094	*P. abies*	1,094
	Abies	168	*A. alba*	168
	Pinus	90	*P. sylvestris*	86
			P. cembra	3
			P. strobus	1
	Pseudotsuga	10	*P. menziesii**	10
	Larix	7	*L. decidua*	7
Broadleaves	*Fagus*	458	*F. sylvatica*	458
	Quercus	106	*Q. robur*	49
			Q. petraea	45
			Q. cerris	2
			Q. sp.	10
	Acer	44	*A. pseudoplatanus*	32
			A. platanoides	3
			A. campestre	2
			A. sp.	7
	Fraxinus	13	*F. excelsior*	5
			F. sp.	8
	Sorbus	7	*S. aria*	6
			S. aucuparia	1
	Carpinus	4	*C. betulus*	4
	Tilia	4	*T. cordata*	4
	Betula	4	*B. pendula*	4
	Alnus	3	*A. glutinosa*	3
	Populus	1	*P. tremula*	1

sp. = chronology was determined only to the genus level in the database

* non-native species

Supplementary Table 2: Scenarios of future climate change with global anomalies of air temperature and carbon dioxide concentrations expected at the end of 21st century.

Reference in the manuscript	Scenario	Alias	Temperature anomaly (2080-2100)	CO_2 concentration (2080-2100)
Low-emission	SSP1-2.6	*Sustainability*	1.8 °C	450 ppm
	SSP2-4.5	*Middle road*	2.7 °C	600 ppm
High-emission	SSP3-7.0	*Regional rivalry*	3.6 °C	850 ppm
	SSP5-8.5	*Fossil-fueled development*	4.4 °C	1100 ppm

Supplementary Table 3: Maximum and minimum values of the VS-Lite model parameters permitted during the calibration procedure. See **Figure 6** for explanation of individual parameters within the model workflow.

**Group of parameters**	**Parameter**	**Description**	**Minimum**	**Maximum**
Temperature response	T1 [°C]	Minimum temperature for growth	2	9
	T2 [°C]	Lower threshold of optimal temperature range	9	20
	T3 [°C]	Upper threshold of optimal temperature range	T2+0.5	30
	T4 [°C]	Maximum temperature for growth	max(20; T3)	35
Moisture response	M1 [v/v]	Minimum soil moisture for growth	0.0	0.3
	M2 [v/v]	Lower threshold of optimal soil moisture	M1+0.025	0.5
Phenology	I0 [-]	Start of the integration window in the previous year	January (-12)	November (-2)
	Acor [-]	Autocorrelation multiplier of previous-year growth rates	-0.5	1.5
