# Supplementary material for: Longer growing seasons will not offset growth loss in drought-prone temperate forests of Central-Southeast Europe
Source: Nat Commun. 2025 Oct 29;16:9535. doi: 10.1038/s41467-025-64568-8 (PMC12572185; doi:10.1038/s41467-025-64568-8)
Supplement: Supplementary file 3 — Reporting Summary [file 41467_2025_64568_MOESM3_ESM.pdf]

Corresponding author(s): Jan Tumajer
Last updated by author(s): Aug 27, 2025

Reporting Summary

Nature Portfolio wishes to improve the reproducibility of the work that we publish. This form provides structure for consistency and transparency in reporting. For further information on Nature Portfolio policies, see our Editorial Policies and the Editorial Policy Checklist.

Statistics

For all statistical analyses, confirm that the following items are present in the figure legend, table legend, main text, or Methods section.

n/a	Confirmed	
☐	☒	The exact sample size (*n*) for each experimental group/condition, given as a discrete number and unit of measurement
☐	☒	A statement on whether measurements were taken from distinct samples or whether the same sample was measured repeatedly
☐	☒	The statistical test(s) used AND whether they are one- or two-sided<br>*Only common tests should be described solely by name; describe more complex techniques in the Methods section.*
☐	☒	A description of all covariates tested
☐	☒	A description of any assumptions or corrections, such as tests of normality and adjustment for multiple comparisons
☐	☒	A full description of the statistical parameters including central tendency (e.g. means) or other basic estimates (e.g. regression coefficient) AND variation (e.g. standard deviation) or associated estimates of uncertainty (e.g. confidence intervals)
☐	☒	For null hypothesis testing, the test statistic (e.g. *F*, *t*, *r*) with confidence intervals, effect sizes, degrees of freedom and *P* value noted<br>*Give P values as exact values whenever suitable.*
☒	☐	For Bayesian analysis, information on the choice of priors and Markov chain Monte Carlo settings
☐	☒	For hierarchical and complex designs, identification of the appropriate level for tests and full reporting of outcomes
☐	☒	Estimates of effect sizes (e.g. Cohen's *d*, Pearson's *r*), indicating how they were calculated

Our web collection on statistics for biologists contains articles on many of the points above.

Software and code

Policy information about availability of computer code

Data collection	Tree-ring width data: PAST4 (SCIEM), TSAP-Win (RINNTECH), WinDendro (Regent Instruments), CooRecorder (Cybis Elektronik & Data AB)<br>Dendrometer data: Mini32 (EMS Brno), Lolly Software (TOMST)
Data analysis	R version 4.2.2. (opensource), R-Studio (opensource), R-packages 'dplR', 'mgcv', 'dendRolAB', and 'ggplot2'<br>Code for the VS-Lite model is available from https://doi.org/10.5281/zenodo.16931899

For manuscripts utilizing custom algorithms or software that are central to the research but not yet described in published literature, software must be made available to editors and reviewers. We strongly encourage code deposition in a community repository (e.g. GitHub). See the Nature Portfolio guidelines for submitting code & software for further information.

Data

Policy information about availability of data

All manuscripts must include a data availability statement. This statement should provide the following information, where applicable:
- Accession codes, unique identifiers, or web links for publicly available datasets
- A description of any restrictions on data availability
- For clinical datasets or third party data, please ensure that the statement adheres to our policy

The main inputs and outputs of the wood formation model have been deposited in the Zenodo database under accession code https://doi.org/10.5281/zenodo.16931899. Main raw tree-ring width series are deposited in the TreeDataClim database (https://treedataclim.cz/en/database/; including sites from the

CzechTerra) and RemoteForests database (www.remoteforests.org). The current climatic niche presented in Fig. 1b is based on chorological data available from https://data.mendeley.com/datasets/hr5h2hcgg4/6. Historical climate from the E-OBS gridded dataset was accessed from https://surfobs.climate.copernicus.eu/dataaccess/access_eobs.php#datafiles and climate anomalies from CMIP6 from https://climateknowledgeportal.worldbank.org/netcdf-browser?prefix=data/cmip6-x0.25/. NDVI data were accessed through https://lpdaac.usgs.gov/products/mod13q1v061/. Following software was used for data collection and preprocessing: PAST4, TSAP-Win, WinDendro, CooRecorder, Mini32, Lolly Software. Statistical analyses were performed in R 4.2.2. using packages 'dplR' (processing of dendrometer data), 'dendRolAB' (non-stationarity test), 'mgcv' (generalized additive models), and 'ggplot2' (charts plotting). Source data are provided with this paper.

Research involving human participants, their data, or biological material

Policy information about studies with human participants or human data. See also policy information about sex, gender (identity/presentation), and sexual orientation and race, ethnicity and racism.

Reporting on sex and gender	*Use the terms sex (biological attribute) and gender (shaped by social and cultural circumstances) carefully in order to avoid confusing both terms. Indicate if findings apply to only one sex or gender; describe whether sex and gender were considered in study design; whether sex and/or gender was determined based on self-reporting or assigned and methods used. Provide in the source data disaggregated sex and gender data, where this information has been collected, and if consent has been obtained for sharing of individual-level data; provide overall numbers in this Reporting Summary. Please state if this information has not been collected. Report sex- and gender-based analyses where performed, justify reasons for lack of sex- and gender-based analysis.*
Reporting on race, ethnicity, or other socially relevant groupings	*Please specify the socially constructed or socially relevant categorization variable(s) used in your manuscript and explain why they were used. Please note that such variables should not be used as proxies for other socially constructed/relevant variables (for example, race or ethnicity should not be used as a proxy for socioeconomic status). Provide clear definitions of the relevant terms used, how they were provided (by the participants/respondents, the researchers, or third parties), and the method(s) used to classify people into the different categories (e.g. self-report, census or administrative data, social media data, etc.) Please provide details about how you controlled for confounding variables in your analyses.*
Population characteristics	*Describe the covariate-relevant population characteristics of the human research participants (e.g. age, genotypic information, past and current diagnosis and treatment categories). If you filled out the behavioural & social sciences study design questions and have nothing to add here, write "See above."*
Recruitment	*Describe how participants were recruited. Outline any potential self-selection bias or other biases that may be present and how these are likely to impact results.*
Ethics oversight	*Identify the organization(s) that approved the study protocol.*

Note that full information on the approval of the study protocol must also be provided in the manuscript.

Field-specific reporting

Please select the one below that is the best fit for your research. If you are not sure, read the appropriate sections before making your selection.

☐ Life sciences ☐ Behavioural & social sciences ☒ Ecological, evolutionary & environmental sciences

For a reference copy of the document with all sections, see nature.com/documents/nr-reporting-summary-flat.pdf

Ecological, evolutionary & environmental sciences study design

All studies must disclose on these points even when the disclosure is negative.

Study description	We used the VS-Lite model of intra-annual wood formation to predict future shifts in growth phenology and kinetics in temperate forests towards the end of the 21st century. We calibrated the model for the 1961-2020 period using a network of 2,013 tree-ring width chronologies from 15 Palearctic tree genera from Central, Eastern and Southeastern Europe distributed from dry lowlands to treelines. To independently validate simulated intra-annual growth patterns, we compared these simulations to 83 direct annual wood formation observations recorded by dendrometers and series of NDVI. We then applied the calibrated VS-Lite model to climatic projections towards the 2100s under four Shared Socioeconomic Pathways to simulate shifts in phenology and growth kinetics under varying rates of climate change and increasing frequency and intensity of stochastic climatic extremes.
Research sample	Time series of inter-annual (tree-ring width chronologies) and intra-annual (dendrometer records) growth dynamics of stems of Central European tree species. The distribution of individual taxa included in our dataset is provided in Table S1 and the spatial pattern is shown in Figure 1 and Supplemetary Figure 15. We aimed to build a robust dataset to characterize growth dynamics of dominant tree species of temperate forests in Central, Eastern, and Southeastern Europe.

Sampling strategy	Details about the sampling strategy at each site - including the number of sampled trees, selection of trees for sampling, and number of cores per tree - are provided as metadata at the site level in the TreeDataClim repository (https://treedataclim.cz/en/database/) or are available from the RemoteForest database (https://www.remoteforests.org/). Only sites with minimal replication of five trees over the full 1961-1995 period were considered. Minimum sample depth is a standard dendrochronological threshold to amplify shared (i.e., climatic) sources of inter-annual variability in mean site chronology.<br>Dendrometers were mounted on tree stems preferentially avoiding injured or declining individuals.
Data collection	Increment cores were dried, mounted on wooden holders, and sanded to improve the visibility of tree-ring borders. Measurements of tree-ring widths were performed using a stereomicroscope or high-resolution flatbed scanner with dedicated software.<br>Dendrometer data were regularly downloaded with a typical frequency of once per year. Persons collecting the dendrometer data are listed in the Author contribution statement at the end of the manuscript.
Timing and spatial scale	Tree-ring width chronologies from 1961 to the last year of the given chronology (1995-2020) were used for the calibration of the VS-Lite growth model. By feeding calibrated models with SSP scenarios of future climate change, we predicted mean growth dynamics for each bidecadal period between 2020-2039 and 2080-2099. Dendrometer data consisted of 83 annual measurements of stem size variation in hourly or sub-hourly temporal resolution for individual years between 2012 and 2020.<br>Spatial distribution of our dataset in Central, Eastern, and Southeastern Europe is shown in Figure 1 and Supplementary Figure 15.
Data exclusions	Sites with replication lower than 5 cored trees were excluded from the analysis due to a potentially significant proportion of individualistic noise in mean site chronology.
Reproducibility	All analyses are reproducible and can be applied to the same or different dendrochronological and climatic datasets.
Randomization	10,000 random combinations of VS-Lite parameters inside their ecologically reasonable intervals were generated during the model calibration to find their optimal sets for each site. A considered reasonable interval is provided for each parameter in Table S3. The combination of parameters resulting in the highest correlation between observed and simulated chronologies was kept for further analysis. The function performing this randomization is available through https://doi.org/10.5281/zenodo.16931899
Blinding	Blinding was not relevant, as individuals (=trees) were not subject to any treatment.

Did the study involve field work? ☒ Yes ☐ No

Field work, collection and transport

Field conditions	The dataset comprises 2,013 sites distributed across temperate forests in Central, Eastern, and Southeastern Europe including dry lowland forests and local treelines (elevation range 153-1713 m a.s.l.). Individual sites capture the distribution of 15 regionally dominant tree species. Mean annual climatic water balance, i.e., difference between annual precipitation and potential evapotranspiration, of individual sites varies between -160 mm and +1230 mm.
Location	2,013 sites across Central, Eastern, and Southeastern Europe (Figure 1)
Access & import/export	Local and national laws were followed and the authors had the needed permits to sample tree cores in the study sites or to install dendrometers. Given the size of the dataset, individual numbers of permission certificates are not provided.
Disturbance	The coring holes have a minimum disturbance effect and are typically healed within the single growing season. Before dendrometer installation on taxa with thick bark (e.g., Quercus, Pinus), slight debarking without injuring living tissue was locally performed to ensure smooth contact between the dendrometer and the stem surface.

Reporting for specific materials, systems and methods

We require information from authors about some types of materials, experimental systems and methods used in many studies. Here, indicate whether each material, system or method listed is relevant to your study. If you are not sure if a list item applies to your research, read the appropriate section before selecting a response.

Materials & experimental systems

n/a	Involved in the study	
☒	☐	Antibodies
☒	☐	Eukaryotic cell lines
☒	☐	Palaeontology and archaeology
☒	☐	Animals and other organisms
☒	☐	Clinical data
☒	☐	Dual use research of concern
☐	☒	Plants

Methods

n/a	Involved in the study	
☒	☐	ChIP-seq
☒	☐	Flow cytometry
☒	☐	MRI-based neuroimaging

Plants

Seed stocks

At least one core with a diameter of 0.5 cm was extracted from each stem using increment corer. Wood samples consist mostly of dead plant tissues. No other types of plant tissues, including seeds, were collected.

Novel plant genotypes

Describe the methods by which all novel plant genotypes were produced. This includes those generated by transgenic approaches, gene editing, chemical/radiation-based mutagenesis and hybridization. For transgenic lines, describe the transformation method, the number of independent lines analyzed and the generation upon which experiments were performed. For gene-edited lines, describe the editor used, the endogenous sequence targeted for editing, the targeting guide RNA sequence (if applicable) and how the editor was applied.

Authentication

Describe any authentication procedures for each seed stock used or novel genotype generated. Describe any experiments used to assess the effect of a mutation and, where applicable, how potential secondary effects (e.g. second site T-DNA insertions, mosiacism, off-target gene editing) were examined.
